# Sugarcane extraction machine injuries of the hand

**DOI:** 10.1186/1753-6561-9-S3-A104

**Published:** 2015-05-19

**Authors:** Sharifah Roohi Ahmad

**Affiliations:** 1 Department of Orthopaedics, Universiti Putra Malaysia, Serdang, 43300, Malaysia

## Introduction

Sugarcane juice is popular drink in Malaysia and South Asia bearing in mind the refreshing taste in the scorching heat and humidity. During the fasting month, family-run businesses will open stalls in designated areas (“Bazaar Ramadhan”) to serve the local population that flocks there. Children sometimes are allowed to handle these machines without their parents realising the hidden dangers in the mechanism and the severity of injuries that may be inflicted. This lecture will deal with these injuries that have a special place due to the mechanism, the biological and technical implications and the extensive damage that becomes difficult to repair or reconstruct (similar to the dough sheeter injuries reported in 2005). A management protocol is suggested to maximise the outcome.

## Materials and methods

An overview of hand injuries in the paediatric population in a tertiary hospital sustained during an occupation or at a worksite were collected between the period of 1^st^ June 2003 to 31^st^ May 2006. The sugarcane extraction machine was then identified as a major culprit.

## Results

There were 6 cases involving 4 boys and 2 girls aged between 5 to 18 years. Most of them were helping their parents in a busy sugarcane juice stall during the fasting month. Due to the unusual mechanism of the injury, the patients being novices and the biological nature of the product (high sugar content), the injuries created an interesting mixture of challenges in the management. Most cases sustained severe crushing with avulsion of the soft tissues resulting in unique treatment problems with limited reconstructive options due to the biological (Pseudomonas infection in all cases) and mechanical nature of the injury. A high amputation rate and poor functional outcome resulted. Usage of the Pedicled Posterior interosseous artery flap was found to be particularly useful in delayed reconstruction.

## Discussion

Preventive measures should be undertaken to avoid these disabling hand injuries. Studies have identified some modifiable risk factors for similar injuries. The risk of hand injuries tend to increase when working with equipment, tools, or work piece which is not performing as expected, or when using a different work method to do a task. Apparently, wearing gloves reduced the relative risk by 60% [2]. Other factors contributing to machine related mishaps are doing an unaccustomed task, being distracted, and being rushed. In this instance hypoglycaemia and a less than suitable environment is also at fault.

## Conclusion

The initial management of these injuries is absolutely critical. Referral to a Hand Unit familiar with crush injuries is an asset. Immediate intravenous antibiotics, early thorough debridement followed by meticulous removal of the sugarcane fibres is essential. If tissues are damaged beyond repair, amputation followed by delayed reconstruction is undertaken.

For these teenagers this type of injury obviously will have an adverse impact to their future quality of life. The initial step would be to create awareness amongst physicians and the public regarding this type of injury [3] and introducing preventive programmes. The various strategies include safety designs for the machines, training on proper handling techniques and enforcement of safety regulations and guidelines.
